# Upper extremity deep vein thrombosis in a 25 year old apparently healthy man

**DOI:** 10.4314/pamj.v4i1.53614

**Published:** 2010-01-09

**Authors:** Fitsum Girma

**Affiliations:** 1 All Africa Leprosy, TB Rehabilitation, Research and Training Centre, Addis Ababa, Ethiopia

**Keywords:** Deep vein thrombosis, axillary vein, subclavian vein, Paget-Schroetter syndrome

## Abstract

This case of upper extremity deep vein thrombosis is selected for case report as it is a rare form of deep vein thrombosis without a very well established treatment modality and prognosis. The objective of this study was to report the outcome of a 25 years old male patient with idiopathic upper extremity deep vein thrombosis treated conservatively with low molecular weight heparin (LMWH) and oral warfarin. The data sources used were patient interview, laboratory and radiology investigation results and patient charts. The patient had no apparent recurrence or complication for 3 years except the presence of occasional dull pain over the affected left upper extremity.

## Background

Upper extremity deep vein thrombosis (UEDVT) is an increasingly important clinical entity with potential for considerable morbidity. Pulmonary embolism (PE) is present in up to one-third of patients with UEDVT. When compared with the lower extremities, the venous pathways of the upper extremities are less likely to develop thrombus because of increased flow, gravitation effects and the absence of stasis [1–4].

Deep vein thrombosis (DVT) of the upper limbs can be primary or secondary [1]. Paget-Schroetter syndrome, a primary upper extremity deep vein thrombosis (UEDVT), is a rare form of DVT with an incidence of 2 per 100,000 persons per year [2]. Subclavian/axillary vein thrombosis was first described by Paget and Von Schroetter independently in the nineteenth century. Paget-Schroetter syndrome (Effort thrombosis) most commonly occurs in young healthy men. It appears more often in the dominant limb as it is associated with effort or strenuous activity [3].

## Patient and case report

A 25 year old left hand-dominant man presented with swelling and mild numbness of the left arm of three days duration. Just a day before the onset of the swelling, he carried a heavy television set to his house. He had a history of pleural tuberculosis which was successfully cured after anti-tuberculosis treatment. He denied any past history of surgery and intravenous drug use. No family history of a similar illness was detected. Clinical examination revealed few dilated veins in the arm and the upper left half of the chest, which became more prominent during Valsalva’s manoeuvre. The left upper limb showed normal arterial pulses and there was no neurological deficit or bony injury. A Doppler ultrasonography confirmed thrombosis of the left subclavian/axillary vein. Other investigation results including x-rays of the left shoulder and chest, computerized tomography of the upper chest and complete blood count (CBC) were all normal. The coagulation profile revealed activated partial thromboplastin time (aPTT) and prothrombin time (PT) within normal range while the international normalized ratio (INR) was 1.0.

The patient was treated as an outpatient with low molecular weight heparin (LMWH) for 6 days and then maintained on oral warfarin for 6 months. A Doppler imaging done after the third month of initiation of warfarin treatment revealed complete recanalization of the subclavian/axillary vein and development of collateral circulation. The patient was followed for three years without any apparent complications or relapse except a mild occasional pain on the affected limb.

## Discussion

Patients with Paget-Schroetter syndrome have developed UEDVT after strenuous activity like weight lifting (like in this case) [4]. Clinical presentation of major venous thrombosis in the upper limb usually presents with swelling of the upper limb, prominence of superficial veins and neurological symptoms [5]. The most serious complication of UEDVT is pulmonary embolism occurring in one third of the cases [6]. The diagnosis of UEDVT is confirmed by either duplex ultrasonography or contrast venography [7].

The treatment options for axillary venous thrombosis include conservative therapy with anticoagulants, catheter-mediated thrombolysis and surgical intervention to remove the intravascular clot or to revise the anatomy of the abnormal costoclavicular space. Patients with axillary-subclavian venous thrombosis due to intrinsic damage require only anticoagulation therapy whereas those with extrinsic obstruction may require correction of the surgical pathology as well [8, 9]. An anticoagulation therapy includes heparin followed by oral warfarin for a period of 3 to 6 months keeping the INR level 2.0 to 3.0. The patient was followed for three years without any apparent complications or relapse except a mild occasional pain on the affected limb.

## Conclusion

This case report provides additional evidence indicating that complications in patients with Paget-Schroetter syndrome can be minimized if managed timely and conservatively.

## Consent

Written informed consent was obtained from the patient for publication of this case report. A copy of the written consent is available for review by the Editor-in-Chief of this journal.

## Acknowledgements

I would like to thank the patient for his willingness to report his case to the scientific community.

## Competing interests

No competing interests are involved in this case report.
